# The lived experience of caring for someone with bipolar disorder: A qualitative study

**DOI:** 10.1371/journal.pone.0280059

**Published:** 2023-01-19

**Authors:** Bronte Speirs, Tanya L. Hanstock, Frances J. Kay-Lambkin

**Affiliations:** 1 School of Medicine and Public Health, University of Newcastle, Callaghan, NSW, Australia; 2 School of Psychological Sciences, University of Newcastle, Callaghan, NSW, Australia; University of Pittsburgh, UNITED STATES

## Abstract

Being a close family or friend of someone with bipolar disorder (BD) can lead to experiences of increased stress, anxiety and depressive symptoms related to the burden of caring. However, the lived experience of being a carer for a person with BD has not received significant research attention. This study aimed to gain further insight into the experiences of individuals in an informal caring role for someone with BD and determine what additional information and support these people need to take care of both themselves and the person they are caring for. Fifteen qualitative interviews were carried out with carers discussing their lived experiences with utilising coping strategies and supporting someone with BD. Following the interviews, thematic analysis was used to identify five key themes. These themes were: Separation of the person and the disorder, carer health and coping strategies, unpredictability and variability of symptoms, carer disillusionment and silencing, and story sharing and support needs. Overall, the findings highlighted the need for increased in-person and online support specifically tailored for carers with loved ones experiencing BD.

## Introduction

In Australia, it is estimated that 1.3% of the population will experience bipolar disorder (BD) in their lifetime [1]. The cyclical nature of BD means that individuals can shift from mania or hypomania phases, characterised by higher energy and less need for sleep, into depressive phases of low interest and motivation [2]. It is a condition that not only presents significant challenges for the individual diagnosed but also has an indirect burden on their carer’s mental and physical health [3]. Perlick et al. [4] reported that 93% of BD carers had moderate or higher distress in at least one domain related to caregiving. In this context, a carer is defined as an individual who provides non-professional and informal personal care, support and assistance [5]. Carers are the primary support person for the individual and may include a spouse, parent, friend, sibling, foster carer or relative who is their main support person [6].

The cyclical and unpredictable aspects of BD can mean that the stressors facing carers can change quite frequently, [7] and this can impact the carers’ overall quality of life [8]. Existing research does not specifically address the caring experience in BD, but instead focuses more broadly on carers of people with mental health disorders [8–10]. Informal carers of people with schizophrenia [9] have reported that caregiving had an emotional, financial, physical and social impact on their lives, as well as difficulties with managing difficult symptoms such as violence. Qualitative interviews [4, 10] with carers of people with serious mental illnesses, including those with BD and schizophrenia, identified carer isolation and stigma, and the need for carers to be more included in decision-making. Informal carers of people using methamphetamine also reported experiencing stigma, which affected them in reaching out for both professional support and support from close family and friends [11].

Whilst there is some international research [12–14] on the caring experience for BD, there is limited research in the Australian context. A British qualitative study that interviewed carers, service providers and people with BD found that to increase carer inclusion in decision-making and care for people with BD, there needs to be changes to healthcare operational frameworks and policy agendas [12]. Through interviews with carers and therapists, Kargar et al. [13] found that carer burden severity varies and is impacted by individual, social and organisational factors. Additionally, a qualitative study with informal carers conducted in Mexico [14] found that carer burden included: financial status, relationships, relapse anxiety, depressive symptoms and for immediate family members, a fear of developing BD themselves. Together, this indicates a greater need for research to consider more specific condition-focused carer supports and experiences.

Evidence is only beginning to emerge about the lived experience of carers during mania and hypomania phases of BD, which make this caring experience unique from others [3]. Financial stress, which in manic phases of BD can lead to impulsive spending, contributes to the financial pressures of informal carers and can add additional stress to the caring relationship [15]. Individuals with BD have lower employment rates, which can also impact the financial strain and contribute to the stress caused by impulsive spending [16]. In the case where a carer is a spouse, the relationship with the person can be put under pressure and become maritally dysfunctional from excessive spending and financial difficulties, irritability, lack of affection from their partner [17, 18] and changes in sexual patterns through different phases of the disorder [19]. An increase in risky behaviours, including alcohol and other substance use, can also occur for individuals with BD during manic phases [15, 20]. This contributes to the stress felt by carers and can leave them feeling worried about the safety of the person and embarrassed by the person’s actions around others [20]. Although not commonly recounted in the literature, Maskill et al. [21] described some aspects of being an informal carer for someone with BD including increased compassion. During depressive or mixed stages- where the person experiences both depressive and mania symptoms at the same time, people with BD are at an increased risk of suicidality [22] and hospitalisation [23], which can be distressing and worrying for carers, further complicating the informal care and support they can provide.

In the Australian context, 72% of informal primary carers are female [24]. As a result, the burden of informal caring is heightened for women. Carers have a vital role in the health outcomes of people with BD and in maintaining their safety [25], which can be restricted when carers are experiencing increased stress and burnout [26, 27]. Carers of people with BD report increased psychiatric symptoms, including stress, anxiety and depression, with the most robust evidence being for depressive symptoms [27]. Perlick et al. [16] found that perceived stigma was positively associated with the presentation of depressive symptoms in carers of people with BD and that 63% of this association was related to reduced social support and coping effectiveness. Further, Perlick et al. [28] found that in informal carers of people with BD, caregiving burden is a strong predictor for the development of carer depressive symptoms.

Cuijpers and Stam [26] reported that a carer’s ability to cope with the person’s behaviour and worry less could reduce their emotional burnout and overall burden. In addition, providing psychological support to carers indirectly supports the health outcomes of the person they are caring for [7].

The primary focus of interventions and programs for carers of individuals diagnosed with BD has previously been on psychoeducation, both in-person [26, 29–31] interventions through counselling and organisations such as ARAFMI [32], a mental health carer organisation and online programs [33–35]. Whilst psychoeducation has been shown to have some effect on improving the experience and reducing the burden for carers of people with severe mental illnesses post-treatment [36], there are still some concerns in the literature around how long these benefits last [37] and the most effective form of delivery [38]. An American study by Casarez et al. [39] identified through focus groups with spouses and partners of people with BD that mobile health devices may help improve wellbeing through providing information and skills related to communication with both their partner and children, stress reduction, medication management and social isolation. Although online cognitive behaviour therapy (CBT) interventions have been found to be effective for people with BD [40], these programs are yet to be developed for carers.

This study reports on the results of a qualitative study in which semi-structured phone interviews were conducted with family and friends of people with BD. Whilst a few previous studies have been on the experiences of informal carers of people with BD [4, 12–14, 39, 41], overall, there is limited research in this area, especially in an Australian context. Additionally, the qualitative questions in this study were focused not only on carer experiences but also focused on support and coping. The study also included carers in various roles, including parents and children, in addition to partners. This was with the intention of improving future support for carers. There were two aims of the study. Firstly, to gain insight into the experiences of carers as they cope with their loved one’s mental illness, including information around warning signs, coping methods, lifestyle factors and the changes in stressors throughout different phases of the illness. Secondly, the study sought to better understand what help and information carers need to take care of both themselves and the person they are caring for.

## Methods

### Context and study design

This qualitative study was part of a wider mixed-methods study that aimed to better understand the experiences, coping and support of informal carers of people with BD. As part of the wider study, eligible participants completed a 20–30-minute online survey on the REDCap survey platform, which asked for demographic information, including their identifying gender, age and relationship to the person with BD, and also asked about their caring experiences. A phenomenological approach [42] was taken to this qualitative study, where meaning about the experiences of carers of people with BD was sought based on the descriptions provided by people with this lived experience. All procedures were approved by the Human Research Ethics Committee at the University of Newcastle (H- 2020–0436).

### Sampling and recruitment

Participants were recruited purposively through social media advertising. Upon completion of the wider survey on REDCap, all participants were asked if they would like to be contacted for a semi-structured phone interview (present study) regarding their caring experiences. Participants who indicated “yes” provided specific consent related to this study.

Participants were eligible for the study if they were a carer of someone with a DSM-5 diagnosis of BD, aged 18 years and over and currently living in Australia. Participants were excluded if they had insufficient English to provide informed consent and be interviewed. A ‘carer’ was defined as the main support person for the individual and may include a spouse, parent, friend, sibling, foster carer or relative who is their main support person [6].

### Participants

The demographic characteristics of the fifteen participants (final sample) are displayed in Table 1. The participants (*n* = 15) were recruited purposively via a Facebook advertisement as part of a wider study. Overall, seventeen participants opted into the phone interview through REDCap. Of these, two did not reply to the two attempts made to contact them via their preferred contact method and therefore were not interviewed.

**Table 1:** Participant characteristics.

ID	Gender	Age	Country of Birth	Cultural Heritage	Relationship to Person with BD	Type of BD	Alcohol and/or Drug Use by Person with BD
**P1**	Female	39	Australia	No	Wife	BD I	No
**P2**	Male	50	Australia	Cantonese	Father	BD II	No
**P3**	Male	57	Australia	No	Husband	BD I	No
**P4**	Female	25	Australia	No	Daughter	BD II	No
**P5**	Female	34	Australia	Mauritian	Wife	BD II	Yes
**P6**	Female	20	Australia	No	Daughter	DK	Yes
**P7**	Female	40	Australia	No	Mother	BD II	Yes
**P8**	Female	28	Australia	No	Friend/Housemate	BD II	No
**P9**	Female	42	Australia	No	Wife	BD I	No
**P10**	Female	51	Australia	No	Father and brother	DK	Yes
**P11**	Female	58	Australia	No	Wife	BD II	Yes
**P12**	Female	60	Australia	No	Mother	BD I	No
**P13**	Female	54	Australia	No	Mother	BD II	Yes
**P14**	Female	32	Australia	No	Wife	BD II	Yes
**P15**	Female	25	Australia	No	Girlfriend	BD I	Yes

Note: DK = Did not know

### Interview protocol and procedure

After providing consent, BS contacted the participants by their preferred contact method to set an appointment time for the phone interview. At the beginning of the interview, BS obtained verbal consent from participants, discussed confidentiality and explained the study’s overall purpose. Each interview was recorded with the participants’ consent using a voice recorder. Participants were given the option to read and edit their transcript once produced. Each interviewee was given a participant identification number, and personally identified information was kept separate from the transcripts. Access to identifiable data was only given to members of the research team and was stored securely at the University of Newcastle, Australia.

The leading author (BS), a female second-year Clinical Psychology Masters student in partial fulfilment of the requirements for the Master of Clinical Psychology, conducted the interviews. BS had previous research experience in conducting qualitative interviews. Each interview ranged from 21–49 minutes (*M* = 34 minutes). Participants were remunerated with a $20AUD digital gift card following the interview. Once completed, the interviews were transcribed verbatim using the Outscribe transcription service. This external transcriber was bound by a confidentiality agreement, and this information was included in the Participant Information Statement.

The semi-structured interview was based around a set of seventeen broad, open-ended questions (Table 2), designed to understand the experience of the person caring for someone with BD and what may be helpful to them. The questions were divided into different sections, including background information, experiences, coping, further information needed and program development. In addition, the interviewer used prompts to explore the participants’ experiences more deeply. The interview schedule was based on a modified version of the Orford et al. [43] approach, which was previously used to interview family and friends of people using alcohol and other drugs. Where possible, participants were encouraged to discuss their answers in a narrative format and question order was changed and rephrased accordingly. Using this approach, interviewees were asked to talk about their experiences, coping mechanisms, lifestyle factors and social support in a way that was less structured and allowed the interviewee to openly share their experiences and their story [43].

**Table 2:** Interview questions.

Question Number	Questions
**Establishing Rapport and Background**	
**1.**	To begin, can you tell me a little bit about yourself (and the person you are caring for) and why you chose to be a part of this study?
**Experience with Caring for someone with BD**	
**2.**	Can you describe your experience, as a friend or family member of someone with BD?
**3.**	What have you found most difficult about being a carer of someone with BD Are there any symptoms you find more difficult to deal with?
**4.**	Has your experience of caring changed over time?
**5.**	Are there any positive things about being a carer for someone with BD?
**Information**	
**6.**	Reflecting on your experiences, who or what has been useful in helping you understand BD? Have you been provided with resources regarding the diagnosis?
**7.**	What other information might have been helpful for you to have been told or given?
**Coping**	
**8.**	Thinking about being a carer for [NAME], how have you noticed this has had an impact on your own quality of life? Have you noticed yourself feeling burnt out or stressed?
**9.**	Do you feel as though you have experienced any changes in your physical or psychological wellbeing, as a result of caring for [NAME]?
**10.**	Would you say there has been a change in your relationship with [NAME]? (*Note*: *Only relevant to participants who knew the person before they were diagnosed)* or How is your relationship with [NAME]? Do you feel like your relationship is straightforward? Does it change often?
**11.**	What has been the impact of caring on your daily routine, do you find times to do the things you enjoy?
**12.**	How have you have reacted or attempted to cope as a carer for [NAME]?
**Support**	
**13.**	Thinking about your role as an informal carer, would you say that you have felt supported? Who has helped you?
**14.**	Are there times where you have felt alone or isolated as a carer?
**Program Development**	
**15.**	What do you think about an online support program aimed at helping friends and family members caring for someone with BD?
**16.**	What is the number one piece of advice you would give to yourself, looking back on your experience as a carer of someone with BD?
**17.**	What are the best pieces of advice or assistance you have received to date?

### Data analysis

Braun and Clarke’s [44] reflexive thematic analysis framework guided the theme development process. The reflexive approach was chosen because it allowed for a flexible and organic way of exploring participants’ experiences and stories. During the interviewing stage, BS discussed reflections with TH and FKL during regular supervision sessions to guide the meaning-making process. BS took notes in a reflective journal during the interviews, and discussions were had with TH and FKL to further guide this reflection.

Each of the three researchers, BS, TH and FKL, familiarised themselves with the data independently by carefully reading and re-reading the transcripts and coding five transcripts each. Themes and patterns in the data were then identified and discussed. These discussions commenced with a “bracketing” process, whereby BS, TH and FKL first identified expected results or ideas and then discussed putting them aside to focus on the actual lived experience and reflections of the person in the interview. Following each discussion, the authors assessed whether new themes had been identified from the interview discussed and where this had occurred, agreed that more interviews should be conducted.

Once all interviews had been completed, the transcripts were coded in full by BS and collated using the NVivo software to manage the data. The three researchers then decided on the naming and stories related to these relevant themes and developed these into a table. The output from the thematic analysis consisted of several key themes and subthemes. BS added the constructed themes and subthemes into the MIRO online whiteboard. Following further reflection and supervision with TH and FKL, BS re-reviewed the themes at this stage to construct five key themes that were representative of the experiences of carers.

## Results

### Thematic analysis: Overview of themes

Overall, informal carers described that there were many challenges associated with caring for someone with BD, several barriers in accessing support and suggested ways in which future support can be improved. Through the process of analysis, five themes were identified: Separation of the person and the disorder, carer health and coping strategies, unpredictability and variability of symptoms, carer disillusionment and silencing, and story sharing and support needs. The themes identified are displayed in Table 3.

**Table 3:** Themes.

Themes and Descriptors	Participants Coded for Theme	Number of Times Coded
1. Separation of the disorder and the person	P1, P2, P3, P4, P5, P6, P7, P8, P9, P10, P11, P12, P13, P14, P15	15
2. Carer health and coping strategies	P1, P2, P3, P4, P5, P6, P7, P8, P9, P10, P11, P12, P13, P14, P15	15
3. Unpredictability and variability of symptoms	P1, P2, P3, P4, P5, P6, P7, P8, P9, P10, P11, P12, P13, P14, P15	15
4. Carer disillusionment and silencing	P1, P2, P3, P4, P5, P6, P7, P8, P9, P10, P11, P12, P13, P14, P15	15
5. Story sharing and support needs	P1, P3, P4, P5. P7, P9, P10, P11, P13, P14, P15	11

#### 1. Separation of the disorder and the person

Many of the interviewees described their experience of their loved one being diagnosed with BD as a long and complex process. Thirteen of the participants recognised that separating the person from the illness allowed them to maintain a better relationship with the person and preserve their own wellbeing. Carers identified that they needed to “Keep remembering it wasn’t personal” when the individual was experiencing a BD episode and, their behaviour or words were out of character. One participant described the process of separating the illness from his wife as:

“Just see it as the illness, it’s the illness playing up and not the person playing up.” (P3, husband, aged 57 years)

Carers’ responses indicated that understanding the biological nature of the disorder was imperative in separating the person from the illness and having empathy for the experience of the person they are caring for. A partner noted that for her, it was critical to engage in conversation with her boyfriend to understand his experiences, his behaviours and how to best support him:

“Yeah, into their shoe’s kind of thing… creating a dialogue with your partner, or with the person you’re caring for. Asking them, “What is that experience for you? Can you explain how this affects you?”, rather than the way it affects me.” (P15, girlfriend, aged 25)

Whilst carers were able to acknowledge when the behaviours they were seeing were related to the disorder, they also highlighted the significance of maintaining strong boundaries and knowing when to “remove yourself from a situation” when the disorder got in the way of their wellbeing. A few of the carers expressed a sense of grief and confusion related to the diagnosis of their loved one and the process of understanding and separating what parts of a person’s behaviour were the disorder and what parts were the person themselves.

#### 2. Carer health and coping strategies

All of those interviewed conveyed that the caring role has had a detrimental effect on their mental health and quality of life, reporting increased anxiety symptoms, depressive symptoms, stress and carer fatigue. Some of these carers reported the exacerbation of previous mental health issues, and others reported mental health issues that were not previously present. In addition to reporting that caring had an impact on their mental health, carers also reported an effect on their physical health, diet, exercise behaviours and substance use behaviours. One interviewee described the difficulties she had with putting on weight whilst her focus was on caring for her husband and his wellbeing:

“Because of constant exhaustion and everything, I put on weight, not having energy to exercise, comfort eating and, yeah, and the rest of it.” (P9, wife, aged 42 years)

Another participant described how she has turned to alcohol at times to cope:

“So, I’ve gone total down bad dark holes, and then I’m drinking, and just totally like… fully just like I need to block my emotional pain because I don’t have room to deal with my own stuff.” (P15, girlfriend, aged 25 years)

Eight of the carers reported seeking psychological or pharmacological assistance for themselves. For some of the carers, the caring role intensified mental health symptoms that they were already experiencing but for others, it was the first time they had had any mental health issues. In order to maintain their wellbeing and protect their mental and physical health, nine carers reported the importance of self-care and the positive effects of nature, exercise, socialising, creative hobbies, yoga and meditation. The positive effects of self-care are described by one participant:

“I think for me, just managing my general health is really important. One of the main things that I used to manage my anxiety is exercise, so just trying to maintain that routine and maintain that exercise regime and continuing to eat, and eat well, and then, just trying to keep some elements of your life.” (P8, friend/housemate, aged 28 years)

For many participants engaging in self-care was not something that had always come easily but something they had learned to do over time to reduce burnout and stress from the role. Another aspect of self-care that some carers learnt over time to reduce stress and burnout was enforcing boundaries. For example, one interviewee describes the boundaries she and her partner have in place to maintain her wellbeing and a positive relationship:

“It’s like peace of mind as well. He’s not allowed to message me while he’s at work, because that can sometimes, I don’t know, make him feel, if I don’t write back, or if he’s in a weird state, he will think something else is going on, if he reads my tone the way he wants to read it. It’s like that thing of like somebody else is talking to him that’s not me. So, it’s just a dangerous thing, so we don’t do that.” (P5, wife, aged 35 years)

#### 3. Unpredictability and variability of symptoms

One of the biggest challenges of being a carer of a person with BD was managing the unpredictability and variability of symptoms. Participants reported an extensive range of hard-to-manage symptoms and behaviours including, suicidal ideation, anger, sexual indiscretions, excessive spending and running away. Overall, all fifteen participants used descriptive and metaphorical language to describe the chaotic up-and-down nature of the symptoms of BD. One interviewee described this as:

“Very emotional. Extremely hard to deal with at times. Just a rollercoaster, is probably the best way to describe it. It’s like being on a rollercoaster” (P13, mother, aged 54 years)

Carers described that one of the hardest and most unpredictable symptoms to manage was violence, irritability, anger and the “hatred they can show towards you.” Another symptom reported as having a high burden on carers was managing suicidal ideation and fear of their loved one dying. This fear was often exacerbated by incidences where the person they were caring for had left the house without warning, which was reported by several interviewees, as one husband described:

“She can go up for a walk up to the hospital, or anything up to six kilometres away, trying to get help without telling me. And so, I wake up in the middle of the night and she’s not in bed, not in the house and whatever, and spend half the night looking for her. Then she turns up the next morning.” (P3, husband, aged 53 years)

Carers reported that they experienced higher stress when their loved one would end up hospitalised, with many reporting several hospitalisations. Eight of the participants reported that the person they were caring for had issues with alcohol or other drugs. With one participant expressing that the person would use alcohol/other drugs to self-medicate the symptoms, they were having.

Eleven of the carers described the symptoms of BD having an impact on their relationship. One participant expressed her concerns about her relationship with her partner with BD and stated:

“Because he can’t control himself, I don’t think I should have, I think it would be irresponsible for me to have children with him.” (P5, wife, aged 35 years)

In addition, four interviewees described increased stress related to sexual indiscretions and out-of-character sexual behaviours during mania, with one partner describing her partner becoming romantically involved with someone else whilst in an inpatient unit:

“He kind of started a relationship with someone else while he was in there and he was asked to leave.” (P9, wife, aged 42 years)

Nine carers described the stress related to excessive financial spending and impulsivity with finances. A wife expressed:

“When I look back, she spent $50,000 on shoes within a six-month period.” (P1, wife, aged 39 years)

Participants described that the unpredictability of symptoms led them to constantly be in flight or fight mode, hyper-vigilant, and experiencing increased anxiety around what would come next. One carer expressed:

“I was always on edge, just always ready to, like, go into defensive mode.” (P9, wife, aged 42 years)

Another participant described this as:

“I used to walk around on eggshells, be too afraid to say anything.” (P13, mother, aged 54 years)

Carers explained that over time they were able to adjust, and one way they learnt to better manage the unpredictability of the disorder is to communicate well with each other, as a daughter described:

“Like deal with the uncomfortable situations that are there before it gets worse and it blows up bigger than it should have been.” (P6, daughter, aged 20)

Whilst many of the carers described negative aspects of the unpredictable caring role, six of the carers reported themes related to becoming more empathetic, compassionate, and patient from their caring role. A few carers also reported that they liked parts of the person’s personality that were related to BD, including increased creativity and charisma. A wife described her partner with BD I:

“A bit of a genius really. But, obviously, with that comes a whole bunch of other stuff.” (P1, wife, aged 39 years)

#### 4. Carer disillusionment and silencing

Overall, carers reported feeling disillusioned by their caring role and the lack of support their friends and family, society, and healthcare professionals offered to them. Carers expressed a heightened need for inclusivity in both the intervention and decision-making processes for their loved ones and a desire for an increased awareness of what is going on so they can better support the person they are caring for. Carers reported that they were often disillusioned by advice given to the person with BD by healthcare professionals who often only focused on the person with BD rather than giving advice that was congruent with wider systems in which the person lives. As a wife cited:

“In that first part…she had to look after herself, and do this and do that, and it was all about her. And it was very selfish. And I’m like, you know what? You don’t exist in a bubble. You don’t. You exist in a family and they’re just telling you how to deal with it. You know, like, ‘Make sure you get 15 hours of sleep or whatever.’ Well, that’s great but you live with a family of people. You know, the house can’t stay silent. We can’t all just walk on eggshells for the rest of our life because you need, you know, I think they don’t tell bipolar people enough how to exist in a world.” (P1, wife, aged 39 years)

Carers discussed how their needs were often "forgotten” and “disappear,” with the primary focus in healthcare being on the person with BD. Additionally, interviewees expressed concern regarding the missed opportunities for healthcare staff to offer support and check in on their wellbeing, as one individual described:

“I go to all of her psychiatry appointments…I’m genuinely surprised that there was no mention or even a question of, how are you coping? Are you doing okay? Is there any supports that we can give you? That was really quite shocking. Like I said, if that person supporting them isn’t doing the best or doesn’t know how to handle the circumstance, it’s like a recipe for disaster” (P14, wife, aged 32 years)

Many carers were also frustrated with the healthcare system, citing issues regarding medication, poor treatment, policy issues, wrong diagnoses, and the cost. Thirteen of the interviewees discussed themes related to carer silence, feeling “invisible,” and not discussing their experiences with others. This was related to experiences of stigma, unhelpful responses from family and friends and guilt related to discussing the diagnosis with others. A daughter of someone with BD expressed the battle she faced in wanting to talk about her experiences but also keep the confidentiality of the person with BD:

“That was the hardest to navigate when I was younger. I didn’t quite understand how to talk about it with anyone else in a confidential way. Yeah, I think the confidentiality thing has been tricky as well… finding it hard to talk to anyone else about it besides close family members can be a bit isolating.” (P4, daughter, aged 25 years)

Another daughter of someone with BD described that her parents had not openly talked to their children about the parents’ diagnosis as she described:

“I think they tried to be really sheltered from me but like I’ve heard stories.” (P6, daughter, aged 20 years)

A few participants explained that when they had told family and friends in the past, they had unhelpful responses that deterred them from discussing with other people in the future. As one partner explained:

“I learnt to just totally divulge that information with the people that I trusted.” (P14, wife, aged 32 years)

Some interviewees whose parents had BD experienced changes in their roles and increased responsibilities. A daughter described a role reversal between herself and her father, who had BD,

“Yeah, yeah it’s like he’s meant to be the father figure but kind of like I’m better at helping myself than he is so yeah.” (P6, daughter, aged 20 years)

Ten interviewees discussed themes related to the stigma of the disorder experienced by both the person with BD and their carer. A mother expressed her experiences with stigma:

“This bizarre illness that is harder to talk about with people. You don’t just bring it up over dinner with his family and say … he was very manic yesterday, and he did something weird. Whereas if he had something else, like diabetes…that would be more reasonable to talk about, and his family’s really weird about mental illness.” (P5, wife, aged 35 years)

Due to the stigma and isolating nature of the caring role, many of the participants reported that it was even more important for people to reach out and seek support.

#### 5. Story sharing and support needs

Eight of the carers reported themes around their desire to want to help other carers at the beginning of their caring journey and advocate for better support and policy changes, with one mother stating:

“I’ve written to the Shadow Health Minister, I’ve done so many things, though I don’t see anything changing quickly so I feel that it’s my responsibility to help where I can…To try and help other people as well.” (P7, mother, aged 40 years)

Each participant had a different set of experiences with their caring role, one interviewee articulates this in her simple but profound statement:

“Yeah, because everybody’s story will be different and experience will be different as well.” (P10, father and brother, aged 51)

All participants endorsed the need for further support for carers and their families, with some participants describing the need for financial support, others the need for online support and some for in-person support. Many participants expressed that support should include other carers and their experiences, as it would help reduce the isolation they feel. One carer expressed that peer support would be most beneficial with other carers of people specifically with BD, describing her experiences with a support group:

“I went for my first session, but they were all women over 60 whose husbands has Alzheimer’s so I couldn’t relate. There was no, it just wasn’t helping me at all, it was just awful.” (P7, mother, aged 40 years)

Fourteen participants mentioned a need for greater practical support, including information about medication, emergency information, the types of BD, symptoms of BD, how to talk about BD with children and practical money tips. Many of the participants had to seek out more information themselves via the internet, as one interviewee explained:

“Everything we know about it; we’ve looked up ourselves.” (P5, wife, aged 35 years)

Fourteen carers reported that it would be beneficial for a support program to include coping strategies and psychological skills, including managing stress, how to react in different situations, self-care ideas and enforcing boundaries. For example, a coping strategy one interviewee described utilising was worry-time:

“I’ve gotten really good at learning to compartmentalise stuff. When you’re in this position you need to learn to do that. So, I set aside times …if she’s in a bad way or I see the warning signs that she’s on her way… I assign myself a certain amount of time to worry.” (P14, wife, aged 42 years)

Six of the carers discussed themes related to the affordability of support and the need for financial support as well, as a partner described:

“Like my dad paid for us to up our private health insurance so that he could go straight in. Yeah, my concerns about accessibility for people, it’s like we don’t have a lot of money but what do people who have less than us do? And that’s really concerning.” (P9, wife, 42 years)

## Discussion

This study aimed to gain further insight into the experiences of informal carers of people with BD and to understand what additional support and information they need for both their own self-care and the care of the person with BD. Overall, the findings show the chronicity and complexity of the role of being an informal carer of someone with BD and the burden this role can have on an individual. The findings also highlight the need for increased practical, online, psychological and financial support for informal carers of someone with BD.

BD is a cyclical condition that is both chronic and relapsing making, the stressors both long-term and acute [2]. As such, some of the stressors facing carers can be unpredictable and change quite quickly [7]. A central way in which the experiences of people with BD diverged from the experiences of carers of people with other mental health issues [45] and carers of people with a chronic or acute health condition [46–49] was in the unpredictability and variability of symptoms. Whilst there were a lot of common themes discussed by each interviewee in our study, the pattern of these themes discussed varied for each person, highlighting the unique story and experience of each carer. This fits with previous international findings by Kargar et al. [13], that carer burden and experiences can be influenced by a range of different individual, social and organisational aspects. This is an important consideration because it means that the experiences, support and needs of each carer will be different, and any intervention will need to be tailored to the needs of the carer and the person they are caring for. It also indicates that any practical information and psychoeducation should cover the various needs and experiences of carers.

BD is a condition that is highly stigmatised compared with more common mental illnesses, including depression and anxiety [16]. The stigma surrounding BD and the associated shame and guilt felt by carers when discussing their loved one’s mental health with others means that a lot of the interviewees have not openly discussed their experiences before, either formally or informally. In comparison to people with a less stigmatised mental health disorder [45] or a health condition [46–49], carers of people with BD experience greater stigma and a greater reluctance to discuss their experiences. Many of the interviewees reported being silent and not openly discussing their caring experiences and struggles. They also were reluctant to seek out support due to the stigma and negative experiences in the past discussing these issues with family, friends and healthcare providers. Whilst the stigma related to BD is greater than that of other conditions, including depression and anxiety [7], it is similar to carers of people using methamphetamine [11] and carers of people with schizophrenia [9]. As a result, it is expected that the silencing of carers with BD would be similar to carers of people using methamphetamine or with schizophrenia. The lack of awareness of the experiences of BD carers can further perpetuate stigma.

It appeared that the semi-structured interview’s storytelling nature allowed participants to share experiences they had rarely talked about with others. As a result, this may have been therapeutic in nature for the participants in and of itself and is a consideration for those developing support programs for carers in this context. In considering future improvements in the provision of peer support, it would be beneficial in the development of any online program to add a component where participants can share and reflect on their personal story. Additionally, it would be beneficial to see the stories of others to see that their experiences are both similar and unique to other carers of people with BD. In-person support could also benefit from incorporating an opportunity for individuals to share and reflect on their experiences, whether this is in group or one-on-one counselling sessions.

Whilst there has been some previous research completed on the experiences of loved ones and professionals [4, 12–14, 39, 41] caring and supporting people with BD, the body of research overall is limited especially, in an Australian context. This study provides insights not only into the experiences of carers but also explores coping mechanisms, barriers and further support that has important insights for the implementation of future support for carers. One of the strengths of this study is that the interviewees are from a wide range of ages (20–60 years), a range of different caring roles, including partners, children and parents and there is a mixture of informal carers for both people with BD I and BD II. As such, the data captures a broad range of themes related to caring experiences. The qualitative nature of the study provides an in-depth insight into the experiences of people caring.

An important research implication that emerged through this study, was that whilst there were broad similarities in experiences, the experience of each carer was distinctly unique and complex. Whilst this study is representative of a broad range of experiences including, type of BD, gender of carer, caring role, time since diagnosis and age of carer, it may be beneficial for future research, to focus on narrower experiences to better understand differences in experiences. For example, further research could focus specifically on one type of carer relationship, for example, solely focusing on romantic partners. Overall, there is a need for research to consider more specific condition-focused supports for carers.

### Clinical implications

Whilst women are overly represented in the interview participants, this is reflective of the increased role women have in caring roles more broadly in society [22]. It also indicates the increased need to support female carers and reduce the discrepancy of the burdens of caring. Many participants reported that they were never offered support or comprehensive psychoeducation by healthcare providers when they were able to attend appointments with the person they were caring for, and few were included in appointments. This highlights the need for increased awareness of the support needs of BD carers and their families by healthcare professionals. As such, BD carers should be offered psychological support and psychoeducation by healthcare providers, including carer services such as ARAFMI [30]. Carers reported difficulties with treatment plans and suggestions from healthcare professionals being impractical for the wider needs of the family “unit” and ignoring responsibilities and roles the person with BD had, for example, as a parent. As a result, in the provision of support, it would be helpful to consider the greater “unit” or system in which the person with BD operates when making suggestions.

Overall, the themes reported by participants in this study were consistent with previous findings by Chatzidamianos et al. [12], that whilst relatives’ involvement in care benefited consumers, relatives and the health system, notable barriers including accessibility and communication often prevented this. The experiences reported were also consistent with previous qualitative findings by Vargas-Huicochea et al. [14], that a carer-integrated approach is needed, to improve the experiences of both the person with BD and their carer. Specifically, the findings indicate the greater need to include informal carers of people with BD in the treatment process as well as in decision-making. This is in line with previous qualitative interviews with informal carers of people with chronic health conditions including, stroke [46], dementia [47], heart disease [48] and cancer [49] and other mental health conditions [45].

Web-based interventions, in general, are cost-efficient and effective [50, 51] and would prove an affordable option for carers of people with BD who may not seek out in-person treatment for themselves. Berk et al. [2] have developed a comprehensive online guide for carers that was informed by a Delphi study including consumers, carers and clinicians [33–35]. However, there remain gaps in services for an online CBT program for carers of someone with BD. Online CBT interventions have been found to be effective for people with BD [40] but have not yet been developed for carers. There is some evidence [52, 53] that online interventions for carers of mental health disorders can be effective, engaging and acceptable. A critical consideration for program delivery is that carers of people with BD have been found to access primary care services more than mental health services for their psychiatric symptoms [54]. Another consideration is that due to the nature of BD stigma, it is essential that future support consider options for support from people with similar experiences.

When a carer has increased wellbeing and reduced mental health symptoms, it increases their ability to care for the person with BD, which will help improve their BD symptom management [25]. As such, carers play a very important role in the treating team for someone with BD. Interviewees identified that it was important for them in managing their relationship with the person, to be able to separate the person from the disorder. Consequently, informal carers would benefit from clinicians providing increased psychoeducation around to help them to be able to identify what behaviours are related to the symptoms.

### Limitations

A limitation of this study was that people who could not read English were excluded from participation. As stigma can be influenced by culture [20], this data and the experiences of caring may not be representative of all cultures. The study is also limited to an Australian-only population and did not include anyone who identified as Aboriginal or Torres Strait Islander.

## Conclusions

Overall, this study revealed the complexity of being an informal carer for someone with BD. The qualitative findings emphasise that the cyclical and unpredictable aspects of BD can mean that the stressors facing carers change quite often and therefore lead to carers experiencing hypervigilance and anxiety related to what is coming next. This unpredictability impacts their burden and contributes to feelings of exhaustion and anxiety. The findings also highlight that stigma related to BD and associated shame and guilt mean that carers are often silent about their experiences and do not often talk to others about them. Additionally, in the qualitative interviews, carers indicated that they were disillusioned with current support and that there were many challenges still present in access to support and reducing carer burden. This indicates a need for greater awareness of the need for carer support.
